# Fully automated segmentation of the left atrium, pulmonary veins, and left atrial appendage from magnetic resonance angiography by joint‐atlas‐optimization

**DOI:** 10.1002/mp.13475

**Published:** 2019-03-22

**Authors:** Menyun Qiao, Yuanyuan Wang, Floris F. Berendsen, Rob J. van der Geest, Qian Tao

**Affiliations:** ^1^ Biomedical Engineering Center Fudan University Shanghai 200433 China; ^2^ Department of Radiology Leiden University Medical Center Leiden 2300 RC The Netherlands

**Keywords:** atrial fibrillation, left atrium, magnetic resonance angiography, multiatlas

## Abstract

**Purpose:**

Atrial fibrillation (AF) originating from the left atrium (LA) and pulmonary veins (PVs) is the most prevalent cardiac electrophysiological disorder. Accurate segmentation and quantification of the LA chamber, PVs, and left atrial appendage (LAA) provides clinically important references for treatment of AF patients. The purpose of this work is to realize objective segmentation of the LA chamber, PVs, and LAA in an accurate and fully automated manner.

**Methods:**

In this work, we proposed a new approach, named joint‐atlas‐optimization, to segment the LA chamber, PVs, and LAA from magnetic resonance angiography (MRA) images. We formulated the segmentation as a single registration problem between the given image and all *N* atlas images, instead of *N* separate registration between the given image and an individual atlas image. Level sets was applied to refine the atlas‐based segmentation. Using the publically available LA benchmark database, we compared the proposed joint‐atlas‐optimization approach to the conventional pairwise atlas approach and evaluated the segmentation performance in terms of Dice index and surface‐to‐surface (S2S) distance to the manual ground truth.

**Results:**

The proposed joint‐atlas‐optimization method showed systemically improved accuracy and robustness over the pairwise atlas approach. The Dice of LA segmentation using joint‐atlas‐optimization was 0.93 ± 0.04, compared to 0.91 ± 0.04 by the pairwise approach (*P* < 0.05). The mean S2S distance was 1.52 ± 0.58 mm, compared to 1.83 ± 0.75 mm (*P* < 0.05). In particular, it produced significantly improved segmentation accuracy of the LAA and PVs, the small distant part in LA geometry that is intrinsically difficult to segment using the conventional pairwise approach. The Dice of PVs segmentation was 0.69 ± 0.16, compared to 0.49 ± 0.15 (*P* < 0.001). The Dice of LAA segmentation was 0.91 ± 0.03, compared to 0.88 ± 0.05 (*P* < 0.01).

**Conclusion:**

The proposed joint‐atlas optimization method can segment the complex LA geometry in a fully automated manner. Compared to the conventional atlas approach in a pairwise manner, our method improves the performance on small distal parts of LA, for example, PVs and LAA, the geometrical and quantitative assessment of which is clinically interesting.

## 1 Introduction

Atrial fibrillation (AF) is the most common cardiac electrophysiological (EP) disorder worldwide. AF commonly originates from the left atrium (LA), a complex anatomical structure with large variation in shape and size. Studies have shown that ectopic beats of AF often have the origin from within the pulmonary veins (PVs) that are connected to the LA chamber.^1^, ^2^ The left atrial appendage (LAA), a vestigial extension from the LA chamber, is reported to be linked to the thromboembolic risk in AF patients.^3^

Circumferential PV ablation is widely performed to treat AF.^2^ The procedure aims to isolate the PVs from the LA chamber, by creating lesions around the PV ostia to block irregular electrical conduction. To reduce the thromboembolic risk in AF patients, the LAA closure procedure is performed to close the LAA ostium, thereby eliminating the LAA from the systemic circulation.^3^, ^4^

Both interventional procedures can largely benefit from prior assessment of the patient‐specific LA anatomy, including detailed geometry of the LA chamber, PVs, and LAA. Reconstruction of their three‐dimensional (3D) geometry enables accurate preprocedural planning, as well as intra‐operative guidance. With development of imaging techniques, the 3D LA geometry can be noninvasively visualized by computed tomography angiography (CTA)^5^ or magnetic resonance angiography (MRA).^6^ In clinical practice, however, viewing the two‐dimensional CTA or MRA image slices while virtually reconstructing the complex 3D geometry of LA chamber, PVs, and LAA is exceedingly challenging even for the most experienced clinicians. Automated computer methods to segment the structures and reconstruct them in three dimensions are highly desirable.

The technical challenges of automated segmentation of the LA lay in the high complexity, irregularity, and variability in its geometry. To segment and differentiate its anatomical parts, namely, the LA chamber, PVs, and LAA, is even more challenging. In literature, a number of automated and semiautomated methods have been proposed to address the segmentation of the LA, which can be categorized into contour‐based, model‐based, and data‐based approaches, that is, the convolutional neural networks (CNN). Ammar et al.^7^ proposed an algorithm based on threshold localization and circularity shape descriptors. Daoudi et al.^8^ proposed a gradient vector flow active contour model. Margeta et al.^9^ proposed an algorithm based on random decision forests learning with thresholded blood pool as initialization. A shape model‐based method was presented by Ref. 10, consisting of statistical shape models and statistical region growing. Zheng et al. proposed model‐based algorithms including a multipart model followed by region growing, using marginal space learning. Besides shape models, atlas‐based models have been used.^11^ Zuluaga et al. and Sandoval et al. proposed algorithms based on multiatlas segmentation propagation.^12^, ^13^ Tao et al.^14^ proposed a fully automatic segmentation method consisting of global localization by multiatlas registration and local refinement by 3D level set. In recent years, CNN shows great potential in segmentation of medical images and has been successfully applied to various anatomical objects.^15^, ^16^, ^17^, ^18^ Xiong et al.^19^ proposed a patch‐based CNN for fully automatic LA segmentation. Mortazi et al. proposed to segment the LA using the encoder–decoder architecture (U‐NET^20^) in a multiview framework and combined with an adaptive fusion strategy.^21^

Contour‐based approaches are generally sensitive to local image quality, and lacking in global shape constraints. Database approaches are potentially the best‐performing ones, but would demand sufficient annotated data to train the neural network, which is typically characterized by tens of thousands of parameters. In this work, we focus on model‐based approaches, which can produce reliable segmentation using limited annotated data. In particular, we aim to address the “diminishing distal part" problem that we frequently encounter in practice with the conventional multiatlas approach: the extruding part of an anatomical structure, that is, the PV from the LA, often has poor segmentation performance. This is explained by the fact that the result is a consensus of multiple image registration, and the agreement on the distal part usually reduces due to the fact that the border of an object is more difficult to align than the central part.

In the conventional atlas approach, the method first registers a set of images with known labels to the given image one by one (i.e., pairwise), then propagates the labels in these images to the given image, and finally fuses all labels into a final segmentation. The idea behind this is that the high variability in geometry and appearance of the given target image is represented in the set of atlas images. While some atlas images will be registered to the target image more accurately than others, subsequent label fusion by simple majority voting or more advanced methods^22^, ^23^ are found to be robust against unsystematic inaccuracies and can produce a fused segmentation that is typically more accurate than each atlas image individually produces.

Nevertheless, image registration is known to be an ill‐posed and nonconvex optimization problem, which typically becomes harder to solve if the appearance and geometries of the two images are farther apart. As we have observed in our experiences, there is in general no guarantee of registration success for all atlas images as each is individually performed; if some of the registration fails, the accuracy of the final result, especially on the distal part, would be negatively impacted. In this light, if the odds of individual registration failure can be reduced, the accuracy of the multiatlas approach will be systemically improved. Instead of dealing with the possible misregistrations at the fusion stage *post hoc*, we aim to improve the accuracy at the registration stage.

In this work, we present and evaluate a novel method, called joint‐atlas‐optimization, that can systematically boost the accuracy of multiatlas segmentation for complex, irregular, and variable objects such as the LA. The rest of the paper is organized as follows: Section 2 describes the datasets for training and testing, and the reference standard for evaluation. Section 3 presents the joint‐atlas‐optimization method. Section 4 describes the experimental setting and reports the evaluation results. Section 5 gives discussions and conclusions.

## 2 Data and reference standard

We used the LA benchmark database from the MICCAI 2013 challenge^24^ to develop and test our proposed method. We focused on the MRA modality as it is radiation‐free, and can potentially be contrast‐free with noncontrast techniques. Moreover, as MRA generally has lower resolution and poorer image quality than CTA, methods working on MRA can be readily extended to CTA.

The database includes 30 MRA datasets, with using 10 for training and 20 for testing. MR acquisition was performed on a 1.5 T Achieva scanner (Philips Healthcare, Best, The Netherlands). A 3D whole heart image was acquired using a balanced steady‐state–free precession acquisition. The sequence acquired a nonangulated volume covering the whole heart with voxel resolution of 1.25 × 1.25 × 2.27 mm${}^{3}$. Images were acquired during free breathing with respiratory gating at end diastole with ECG gating. Typical acquisition time for a complete volume was 10 min. Each dataset represents a 3D volume image at a single cardiac phase. The datasets were selected to provide a variety of quality levels in the following proportions: nine high quality, ten moderate quality, six local artifacts, and five high noise datasets.

**Figure 1:**
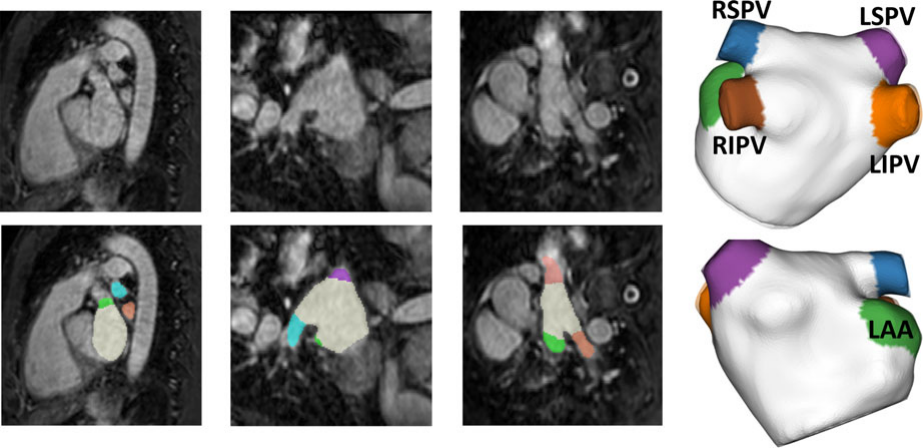
Example datasets as provided by the left atrium (LA) benchmark database: left is the two‐dimensional images, and right is the three‐dimensional mesh. Colour overlay shows the manual ground‐truth, including the LA chamber, pulmonary veins (PVs), and left atrial appendage (LAA). [Color figure can be viewed at http://wileyonlinelibrary.com]

All dataset are provided with LA segmentation as the ground truth (GT). The GT segmentations were started by performing an automatic model‐based segmentation optimized for MRA. After the initial automated segmentation, an experienced observer manually corrected the segmentation whenever necessary. Additionally, a second observer also performed the same manual correction based on initial segmentation to estimate interobserver variability. Ten datasets were selected as the training set, and the other 20 datasets were used for testing. The separate labelling of LA chamber, PVs, and LAA from the segmented LA were realized by the standardized code provided by the challenge organizer. Four labels were given to different PVs: left superior PV (LSPV), left inferior PV (LIPV), right superior PV (RSPV), and right inferior PV (RIPV). An example in the training dataset is shown in Fig. 1, in which 3D visualization of different anatomical labels is given.

## 3 Methods

### 3.A. Multiatlas segmentation

Mutli‐atlas segmentation is a powerful image‐based approach to segment complex anatomical structures in medical images.^25^ Define $A_{i}$ as the atlas image, with known label $L_{i}$, *i* = 1,2,…,*N*, where *N* is the total number of atlases. Multiple images with labels are used as a base of knowledge, to segment a new image *I* containing the same anatomical structure. In the conventional setting, the *N* atlas images are registered to the given image one by one, to derive *N* transformations:

(1) $${\hat{\mu }}_{i}=\text{arg}\min_{\mu_{i}}C\left(T_{\mu_{i}};I,A_{i}\right),\;i=1,2,\ldots ,N$$

where *C* is the cost function for pairwise registration, and $\mu_{i}$ denotes the transformation parameters from the *i*th atlas image $A_{i}$ to the given image *I*. The cost function *C* usually is defined as the mutual information between the pair of images,^26^ a classical registration metric robust to image intensity and modalities. The resulting transformation $T_{{\hat{\mu }}_{i}}$ is used to map the known atlas segmentation to the given image $S_{i}\;=\;T_{{\hat{\mu }}_{i}}\left(L_{i}\right)$, where $T_{{\hat{\mu }}_{i}}\left(\cdot \right)$ denotes the transformation with the optimized parameter ${\hat{\mu }}_{i}$. The *N* segmentations are then fused by the simple majority voting, or other more advanced methods.^22^, ^23^

### 3.B. Joint‐atlas‐optimization

Instead of *N* separate, independent registration processes, we propose to register all atlas images in one registration step to the given image. We formulate it as optimizing a group objective function that merges the given image with the atlas images:

(2) $$\hat{\mu }=\text{arg}\min_{\mu }C(T_{\mu };I,A_{1},A_{2},\ldots ,A_{N})$$

in which the cost function *C* can be defined in various ways^29^, ^30^ as a groupwise registration metric. In this work, we chose to minimize the variance in the group of images:

(3) $$C\left(\mu \right)={\left(T_{\mu }\left(I\right)-{\overset{¯}{I}}_{\mu }\right)}^{2}+\sum_{i=1}^{N}{\left(T_{\mu }\left(A_{i}\right)-{\overset{¯}{I}}_{\mu }\right)}^{2}$$

with ${\overset{¯}{I}}_{\mu }$ named as the “mean‐shape” image:

(4) $${\overset{¯}{I}}_{\mu }=\frac{1}{N+1}\left\{T_{\mu }\left(I\right)+\sum_{i=1}^{N}T_{\mu }\left(A_{i}\right)\right\}$$

where the transformation parameter ***μ*** is the ensemble of all transformation applied to *N* atlas images $A_{i}$ and the given image *I*. For meaningful variance, normalization of image intensity range was applied to each $A_{i}$ and *I* prior to registration.

**Figure 2:**
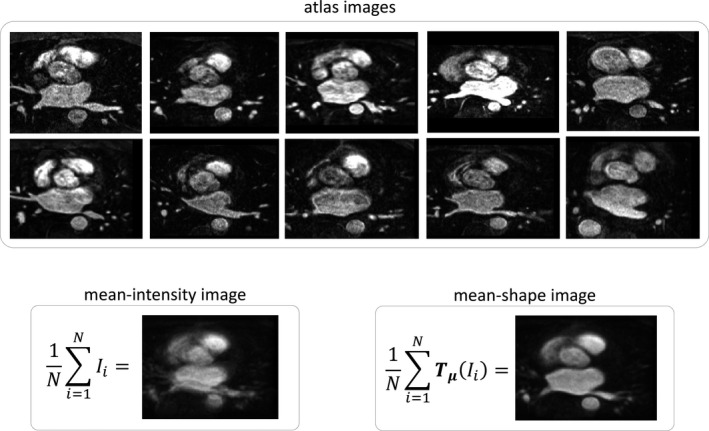
The concept of mean‐shape: the upper panel shows the atlas images in a group, the lower left panel shows the mean‐intensity image of the atlas images, and the lower right panel shows the mean‐shape image of the atlas images. Note that the computation is in three dimension, and for ease of illustration we showed only a two‐dimensional slice containing left atrium.

It can be seen that Eq. (3) essentially represents a groupwise registration,^29^, ^30^ with the given image merged to the atlas images as one group. While in most previous work the groupwise registration minimizes the variance over the time dimension, we minimize the variance over the “atlas‐dimension” along which the given image is also embedded. The resulting mean‐shape image ${\overset{¯}{I}}_{\mu }$ is an average in terms of intensity, and more importantly, it is an average in terms of shape. Figure 2 illustrate the “mean‐shape” concept.

### 3.C. Hierarchical registration

The complexity of the problem requires careful formulation of registration steps. We used a hierarchical strategy: first an affine registration was performed, where *μ* in $T_{\mu }$ contains three translational, one scaling, and three rotational parameters, to roughly align the position, size, and orientation of the objects, then a B‐spline nonrigid registration was performed, where $T_{\mu }$ is defined as:

(5) $$T_{\mu }\left(x\right)=x+\sum_{x\in N_{x}}p_{k}\beta^{3}\left(\frac{x-x_{k}}{\sigma }\right)$$

where $x_{k}$ is the control point, $\beta^{3}$ is the cubic B‐spline polynomial, $p_{k}$ is the B‐spline coefficient vector, *σ* is the B‐spline control point spacing, and $N_{x}$ is the set of all control points within the compact support of the B‐spline at *x*. The control points $x_{k}$ are defined on a regular grid *k*. These parameters form the set of transformation parameters *μ*. The control point grid is defined by the amount of space between the control points $\sigma \;=\;(\sigma_{1},\ldots ,\sigma_{d})$ (with *d* the image dimension). B‐splines have local support ($|N_{x}|$ is small), which means that the transformation of a point can be computed from only a couple of surrounding control points. This is beneficial both for modelling local transformations, and for fast computation.

To solve Eqs. (1) and (2), a stochastic optimization procedure was used.^31^ A multiresolution approach was used to perform the nonrigid registration in a coarse‐to‐fine manner. The same hierarchical strategy and optimization routine can be applied to both pairwise atlas and joint‐atlas registration.

### 3.D. Label fusion on the mean‐shape image

The ten known labels in the atlases were propagated to the mean‐shape image, and fused by majority voting. The inverse transformation from the mean‐shape image $\overset{¯}{I}$ to the given image *I* can be obtained by solving $T_{\mu }^{\prime }\left(T_{\mu }\left(I\right)\right)\;=\;I$.^29^ Subsequently, the segmentation on the mean‐shape image $\overset{¯}{I}$ can be back‐propagated onto the given image *I*.

### 3.E. Local refinement

As demonstrated in the LA Segmentation Challenge,^24^ local contour‐based methods can provide incremental improvement after applying global model‐based methods. Local refinement can adapt the final results to details specific to the given image, especially in irregular parts such as PVs and LAA. With the atlas‐based segmentation as initialization, the level set^32^ was applied with the energy function formulated as:

(6) $$\begin{array}{cc} & F\left(\phi \right)=\mu \int_{\Omega }\left|\nabla H(\phi )\right|d\Omega +\nu \int_{\Omega }H\left(\phi \right)d\Omega \\ & \;+\lambda_{1}\int_{\Omega }{\left|I\left(x,y,z\right)-c_{1}\right|}^{2}H\left(\phi \right)d\Omega \\ & \;+\lambda_{2}\int_{\Omega }{\left|I\left(x,y,z\right)-c_{2}\right|}^{2}\left(1-H\left(\phi \right)\right)d\Omega \end{array}$$

where *ϕ* is the level set function. The model assumes that the image *I* is a 3D image with piecewise constant values. It defines the evolving surface Ω = 0 as the boundary of object to be detected in image *I*. $c_{1}$, $c_{2}$ are the average values inside and outside the boundary, respectively. *H* is the Heaviside function. *μ* ≥ 0,  *ν* ≥ 0, $\lambda_{1},\lambda_{2}\;>\;0$ are weighting factors of the four terms. The first term denotes the surface area, and second term denotes the volume inside the surface. The last two terms are the variance inside and outside the boundary.

**Figure 3:**
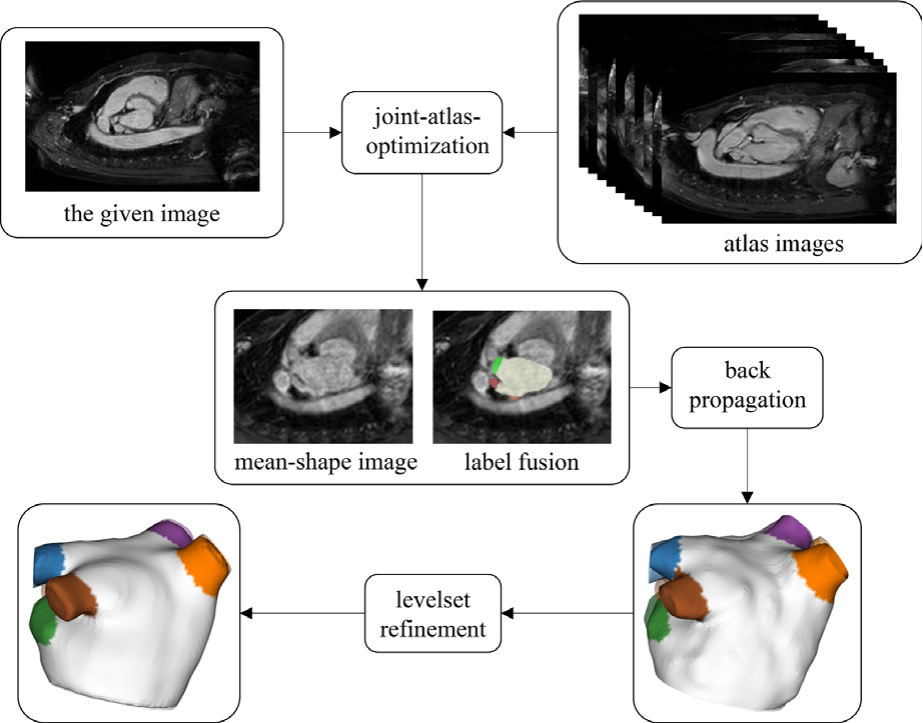
The diagram of the proposed segmentation workflow, including joint‐atlas‐optimization, label fusion on the mean‐shape image, and level set refinement. [Color figure can be viewed at http://wileyonlinelibrary.com]

The complete workflow of the proposed segmentation approach is given in Fig. 3.

### 3.F. Evaluation criteria and statistical analysis

Following the guidelines from the LA benchmark database,^24^ we evaluated the segmentation accuracy using two metrics: surface‐to‐surface distance (S2S) and Dice index. S2S describes the average distance of each point from the automatically segmented surface to the GT surface. Low values of S2S represent high accuracy. We evaluated segmentation accuracy in the LA geometry, as well as in its anatomical parts, namely, the LA chamber, LSPV, LIPV, RSPV, RIPV, and LAA. To evaluate our method on a fair basis against previously published methods, we used the standardized code for computing the S2S distance and Dice index, as well as for differentiating the PVs and LAA. Details of the computation in the evaluation can be found in Ref. 24.

Continuous variables are expressed as mean ± standard deviation. Paired variables were compared using the Student *t*‐test. *P* < 0.05 was considered statistically significant.

## 4 Experiments and results

### 4.A. Experiment setting

To evaluate the proposed joint‐atlas‐optimization method, we also implemented the conventional pairwise atlas approach as a reference. For fair comparison, we used identical registration parameters whenever applicable. In both cases, affine registration was first applied to roughly align objects, followed by a nonrigid B‐spline registration with the same number of pyramids (4) and grid size [*σ* = 10 mm in Eq. (5)]. The same optimization method was used in both cases, and the same fusion method, that is, majority voting, was used. In addition, the same level set refinement was applied afterward. The only difference was that our method performed one single registration to minimize the variance over the atlas‐dimension in the group of 11 images, while the conventional method performed the registration ten times to maximize mutual information in pairs.

All registrations were implemented using the Elastix toolbox^26^, ^33^. We used a spline grid size of 10 mm, three image resolutions, and a fixed number of iterations for each resolution (1000). The variance‐over‐last‐dimension metric was used after normalizing the image intensity to a fixed range of 0–255. The stochastic gradient descent approach was chosen for optimization. Evaluation and statistical analysis were performed in the Matlab environment (R2015b, MathWorks, Natick, MA, USA).

### 4.B. Segmentation accuracy of LA

A visual check showed that all LAs in 20 testing datasets were successfully segmented using the proposed method. Table 1 reports the LA segmentation accuracy in terms of Dice and S2S distance. We listed the results before the level set refinement to show the difference caused by the atlas approach alone, and the results after level set refinement to show the influence of atlas approach on the final results. It can be seen from Table 1 that the joint‐atlas‐optimization approach improved the segmentation accuracy over the pairwise approach both before and after applying the level set.

**Table 1:** The Dice and surface‐to‐surface (S2S) of the left atrium (LA) segmentation results using two different atlas approaches. The Dice and S2S after applying the level set refinement are also reported

	Pairwise atlas	Joint‐atlas	*P*‐value
**Results after atlas segmentation**			
Dice	0.84 ± 0.20	0.92 ± 0.04	0.05
S2S (mm)	2.29 ± 0.79	1.53 ± 0.54	<0.001
**Results after level set refinement**			
Dice	0.91 ± 0.04	0.93 ± 0.04	<0.05
S2S (mm)	1.83 ± 0.75	1.52 ± 0.58	<0.01

The Dice of LA segmentation using joint‐atlas‐optimization was 0.92 ± 0.04 and 0.93 ± 0.04, before and after level set refinement, respectively, while using the pairwise atlas, the Dice was 0.84 ± 0.20 (*P* = 0.05) and 0.91 ± 0.04 (*P* < 0.05), respectively. The mean S2S distance for joint‐atlas‐optimization was 1.53 ±  0.54 mm and 1.52 ± 0.58 mm, before and after level set refinement, while using the pairwise atlas, the mean S2S distance was 2.29 ± 0.79 mm (*P* < 0.05) and 1.83 ± 0.75 mm (*P* < 0.05), respectively.

**Figure 4:**
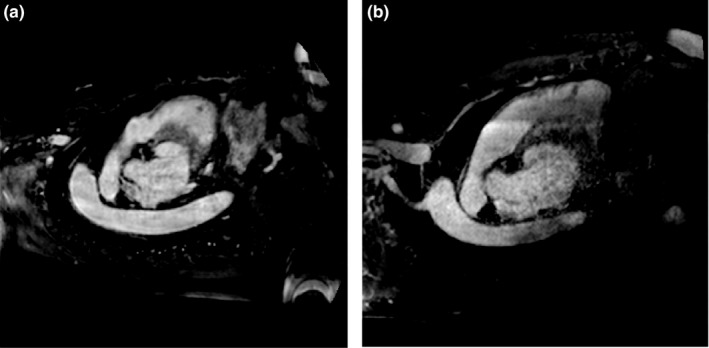
Two examples quantified by the blind/referenceless image spatial quality evaluator (BRISQUE) criterion: (a) an example of high‐quality image(score = 23.15), (b) an example of low‐quality image(score = 48.83).

We further compared the outcome of the LA segmentation between MRA images of different quality levels. The 20 testing data were separated into two groups of ten high‐quality MRA datasets and ten low‐quality MRA datasets by an established image‐quality evaluator: the blind/referenceless image spatial quality evaluator (BRISQUE).^34^, ^35^ The method provides a holistic measure of image quality based on image statistics. The BRISQUE score typically has a value between 0 and 100, with 0 representing the best quality, and 100 the worst.^34^ According to the ranking of BRISQUE scores, we divided the 20 testing data in two groups of 10: high quality and low quality with a threshold of 40. Figure 4 shows two examples quantified by the BRISQUE score. The Dice indices of the high‐quality group were 0.93 ± 0.02, ranging 0.89−−0.96. The Dice indices of the low‐quality group 0.92 ± 0.03, ranging 0.89−−0.95. Unpaired Wilcoxon test showed no significant differences between the two groups (*P* = 0.3). The mean S2S distances were 1.28 ± 0.27 mm for the high‐quality group, and 1.58 ± 0.3 mm for the low‐quality group (*P* = 0.1).

### 4.C. Segmentation accuracy of LA chamber, PVs, and LAA

We also separately evaluated the segmentation accuracy for the LA chamber, four PVs, and LAA, as reported in Table 2. The final results after level set refinement was further compared to the best performance in the LA benchmark.^24^ Interobserver variability as provided by the LA benchmark database is also given. It can be seen from Table 2 that in terms of atlas segmentation, the proposed joint‐atlas‐optimization approach outperformed the conventional pairwise atlas approach in all PVs, while for the LA chamber and LAA the difference was not statistically significant. After applying the refinement, the results were improved for both approaches, but the joint‐atlas‐optimization approach outperformed the pairwise atlas approach consistently given its more accurate initialization. The proposed workflow, that is, joint‐atlas‐optimization followed by level set, outperformed the best in the benchmark. In particular, the standard deviation was lower for all LA components, suggesting more robust performance with MRA data of different quality.

**Table 2:** Experiments on the atlas set to compare the registration strategies (groupwise and pairwise) and the improvement after applying the level set on the image segmentation in terms of the DC of left atrium (LA) body, left atrial appendage (LAA), and pulmonary veins (PVs)

	Pairwise atlas	Joint‐atlas	*P*‐value
**Dice indices after atlas segmentation**			
LA chamber	0.84 ± 0.20	0.92 ± 0.03	0.07
LSPV	0.38 ± 0.16	0.67 ± 0.13	<0.001
LIPV	0.49 ± 0.21	0.68 ± 0.12	<0.001
RSPV	0.17 ± 0.11	0.52 ± 0.17	<0.001
RIPV	0.14 ± 0.13	0.39 ± 0.19	<0.01
LAA	0.81 ± 0.21	0.91 ± 0.03	0.07

	Pairwise atlas	Joint‐atlas	*P*‐value	Best in benchmark	Interobserver
**Dice indices after level set refinement**					
LA chamber	0.90 ± 0.04	0.92 ± 0.03	<0.05	0.91 ± 0.09	0.92 ± 0.04
LSPV	0.50 ± 0.14	0.73 ± 0.15	<0.001	0.72 ± 0.28	0.78 ± 0.03
LIPV	0.59 ± 0.16	0.75 ± 0.12	<0.001	0.76 ± 0.38	0.78 ± 0.04
RSPV	0.28 ± 0.15	0.57 ± 0.19	<0.001	0.35 ± 0.28	0.77 ± 0.03
RIPV	0.26 ± 0.15	0.46 ± 0.17	<0.01	0.46 ± 0.29	0.76 ± 0.04
LAA	0.88 ± 0.05	0.91 ± 0.03	<0.01	‐	0.92 ± 0.03

**Figure 5:**
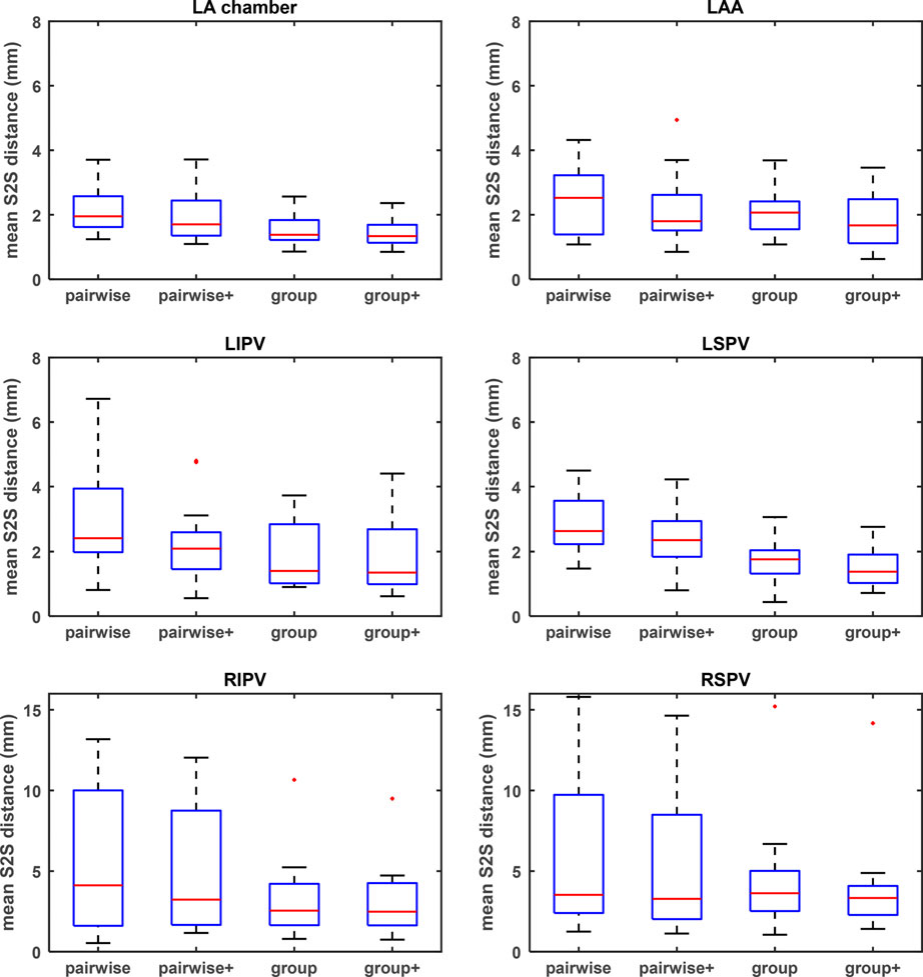
Box‐plots of the mean S2S error for different components of left atrium (LA): LA chamber, left atrial appendage (LAA), left superior pulmonary veins (LSPV), left inferior PV (LIPV), right superior PV (RSPV), and right inferior PV (RIPV). For each component, four methods were compared. (1) pairwise: pairwise atlas, (2) pairwise+: pairwise atlas plus level set refinement, (3) joint: joint‐atlas‐optimization, and (4) joint+: joint‐atlas‐optimization plus level set refinement. [Color figure can be viewed at http://wileyonlinelibrary.com]

Figure 5 illustrates the segmentation performance in terms of mean S2S distance, comparing the joint‐atlas‐optimization and pairwise atlas approaches before and after level set refinement. In all cases, the level set refinement either lowered the mean or standard deviation of the S2S distance, or both. Compared to the pairwise atlas approach, the joint‐atlas‐optimization approach performed consistently better, both before and after the level set refinement.

**Figure 6:**
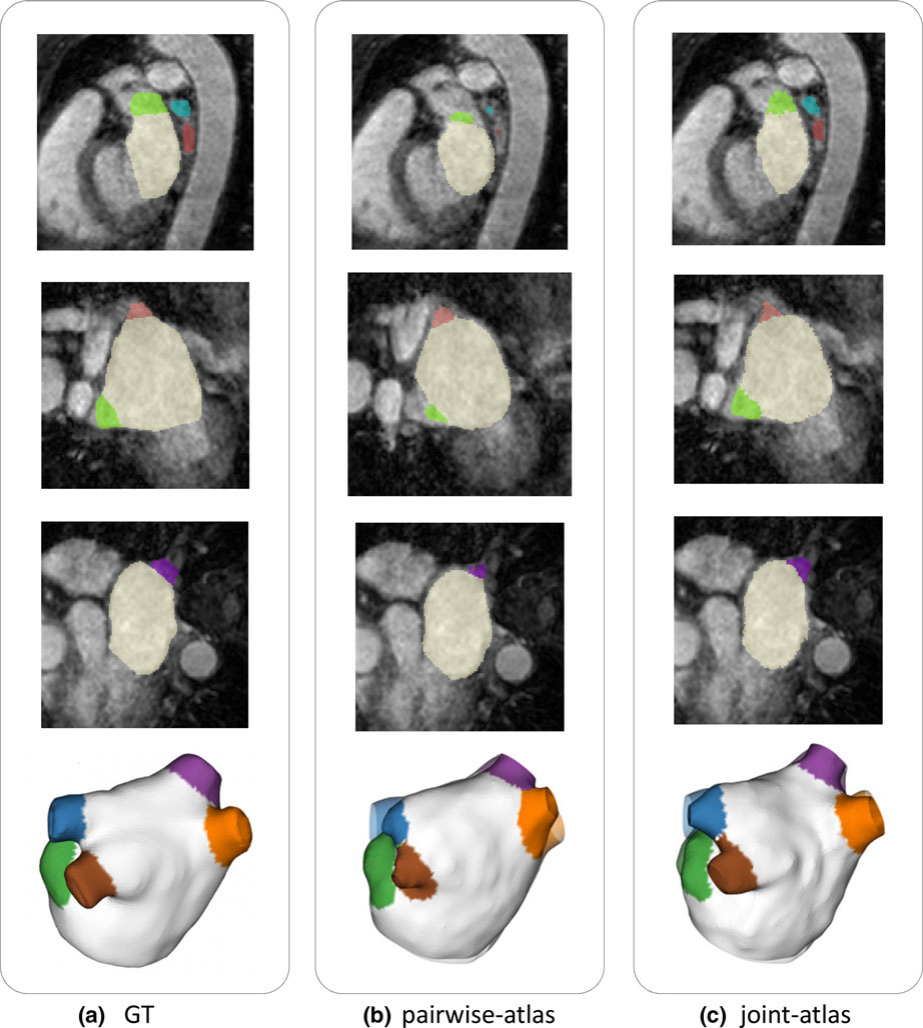
Comparison of the segmentation results before applying the level set refinement: (a) ground truth, (b) pairwise atlas, and (c) joint‐atlas‐optimization. [Color figure can be viewed at http://wileyonlinelibrary.com]

An example is given in Fig. 6 showing the two‐dimensional slices and reconstructed 3D volumes of the GT, pairwise atlas, and joint‐atlas‐optimization segmentation results. The results were the direct outcome of the atlas segmentation, without level set refinement. The proposed approach showed improved segmentation accuracy, in particular in the PVs, producing a better initialization for the level set.

**Figure 7:**
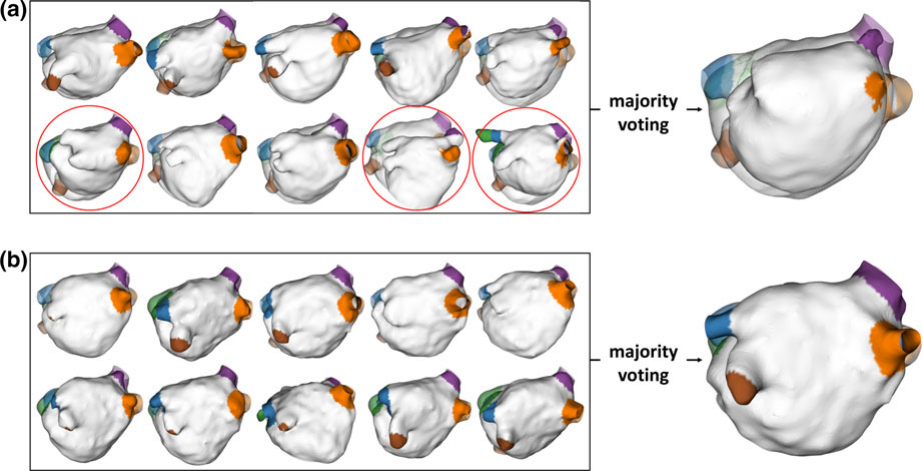
Intermediate registration results of individual atlases by (a) the pairwise atlas approach and (b) the proposed joint‐atlas‐optimization approach. Unsuccessful registrations are marked with red circles. [Color figure can be viewed at http://wileyonlinelibrary.com]

In Fig. 7 we show an example of the intermediate results from the pairwise atlas and joint‐atlas‐optimization approaches. It can be seen that the pairwise atlas, although good enough to localize and segment the complex structure, could fail in individual pairwise registration. Consequently, the “disagreement” of voting is especially pronounced at the small distant part of the structure, that is, the PVs, where the segmentation accuracy was clearly negatively impacted. In comparison, with the cost function optimizing the matching of all atlases, the joint‐atlas‐optimization approach could achieve more consistent registration performance across individual atlases, thereby achieving higher segmentation accuracy, even at the distant part.

### 4.D. Execution performance

For joint‐atlas segmentation, the typical computation time was 554 s, in comparison to 684 s by the pair‐wise atlas method, on a computer with a CPU of Intel Xeon E5‐2687W processor (3.00 GHz) and 64 GB RAM. In both these cases ten atlases were used, and except for the optimization metric, all configurations of registration (e.g., optimization method, grid size, number of iterations, multiscales) were set to be exactly identical.

## 5 Discussion

In this work, we have developed and validated a new method, called joint‐atlas‐optimization, to segment the complex LA geometry from 3D MRA images. The method further differentiates the LA chamber, PVs, and LAA. Trained and evaluated on the public LA benchmark database,^24^ the proposed method showed improved performance over the best in Benchmark in terms of both accuracy and robustness.

The LA is a complex anatomical structure with large variation in shape and size. It is the origin of AF, the most prevalent cardiac disorder worldwide. Clinical studies have shown that different components of the LA likely underlie different aetiological mechanisms. Ectopic beats of AF often originate from the connection between PVs and LA chamber. Myocardial fibrosis on the LA chamber is critically linked to the severity of AF and prognosis.^36^ The LAA, an appended structure to the LA, is associated with the formation of thrombus and hence risk of stroke. Interventional procedures, such as circumferential PV ablation and LAA closure, have been developed to treat AF patients targeting specific anatomical locations. The procedures require careful localization to deliver the therapy (i.e., RF power or clipping). To assess not only the LA geometry but also the separate anatomical parts such as PVs and LAA, is clinically interesting. However, the high complexity, irregularity, and variability of the components put forward high requirements on the accuracy, robustness, and flexibility of the automated algorithm.

Extensive evaluation in the MICCAI LA challenge^24^ has demonstrated the superior performance of the multiatlas approach over other contemporary methods. In most atlas‐based methods, the registration was performed in a pairwise manner. The approach is generally robust to localize the object but can be inaccurate in following fine structures of the object, where the voting is likely to diverge. As observed in our experiments, this is a process with an certain degree of unpredictability: some registration might fail. The failing ones do not destroy the final result, but do negatively impact it accuracy, especially in small distant structures such as the PVs.

**Figure 8:**
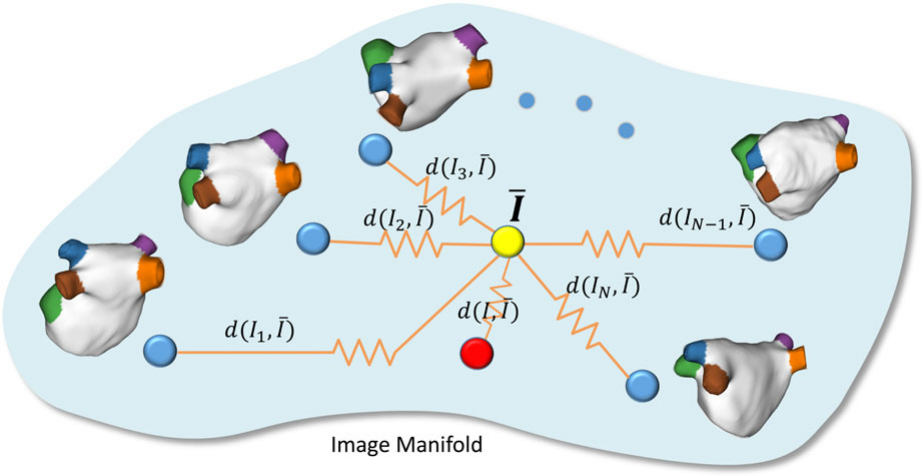
Illustration of the image manifold, in which the atlas images $I_{i}$ (*i* = 1,…,*N*), the given image *I*, and the mean‐shape image $\overset{¯}{I}$ are shown. *d* denotes the distance in the image manifold. [Color figure can be viewed at http://wileyonlinelibrary.com]

In theory, the atlas images, plus the given image, all reside in a complex high‐dimensional image manifold. Depending on similarity between the given image to the individual atlases, the difficulty to register differs. In principle, there are two ways to address the problem: (a) to increase the number of atlases, and to design advanced rules to select suitable atlases or to fuse the labels based on their reliability,^37^, ^38^ (b) to improve the accuracy of atlas registration. The proposed groupwise‐atlas approach falls into the second category, since in many applications as this particular one, the number of available atlases is limited and it is important to make good use of each one. Therefore, we formulated a registration problem between the given image and all atlas images. By merging them into one group, we take advantage of the aggregated information from the point set instead of scattered points in the manifold, as illustrated in Fig. 8. Optimizing the similarities among all images acts as a regularization, making it less likely to be trapped in local minima as it is in the pairwise registration. While an individual atlas image might be too faraway from the given image to register correctly in a pairwise manner, its proximity with other atlas images can help converge them all to a mean‐shape image. The mean‐shape image virtually lies in the middle of the point set, which has an optimized overall distance (hence confidence to register correctly) to all individual points. In this way, we expect to systematically boost the performance of the multiatlas segmentation, and this was demonstrated by our experiment with the LA benchmark database. The method is expected to also work well in similar problems where the anatomical structure is complex and the number of atlases is limited.

From Tables 1 and 2, we have not only observed a significant increase in the average Dice but also a large decrease of the standard deviation, which suggest improved accuracy as well as robustness. In particular, for the PVs, the improvement was pronounced. The underlying mechanism can be appreciated from Fig. 7: compared to the LA chamber, failing cases of pairwise registration has a relatively large impact on the accuracy of distant structures such as PVs; by eliminating the failing cases, our method resulted in a better agreement of the atlases even at the small distant part, leading to a significant boost of the PV segmentation accuracy.

Traditionally we consider improving the segmentation accuracy at the distal part by local methods such as the level set. However, it is known that the level set method, although flexible and attending to details, is sensitive to initialization and tends to be trapped by local noises (as is typical of MRA images). We have observed that the proposed approach still significantly outperformed the pairwise atlas approach after refinement, suggesting the importance of accurate initialization.

In our experiments, we have shown that the proposed joint‐atlas‐optimization approach systematically outperformed the conventional pairwise atlas approach. However, as can be inferred from Fig. 8, the methodology works well only when the atlas images nicely spans the image manifold for the LA. If the atlas images are chosen as such that the given image is far away from all of them, registration failure can occur in a similar way as in pairwise registration. To take advantage of the mutli‐atlas approach, however, it is a general rule that the atlas images should well represent the mean and scattering of the shapes. Another limitation of the work is that in order to evaluate our method against previous methods, we have used the standardized code provided by the LA Challenge database to differentiate the PVs and LAA, while in principle we could differentiate them based on the label values, without postprocessing.

In the past few years, the data‐based CNN segmentation methods have achieved very high performance in LA segmentation, surpassing atlas‐based methods. Xiong et al. used a patch‐based CNN for fully automatic LA segmentation, and achieved a Dice score of 0.940 and 0.942 for the LA epicardium and endocardium, respectively.^19^ The method, however, was data intensive, employing a dataset of 154 AF patients. Mortazi et al. used the encoder–decoder U‐net in a multiview framework, and achieved a Dice value of 0.951 using leave‐one‐out cross validation on the same dataset (29 used for training and 1 used for testing).^21^ Deep‐learning‐based approaches have great advantages in accuracy and speed when there is sufficient training data. Nevertheless, in scenarios where the annotated dataset is scarce and where the training and testing datasets may have different properties, the atlas‐based approach can still be the method of choice to provide reliable and robust segmentation; our proposed joint‐atlas‐optimization further refines the segmentation at the border, producing more accurate anatomical depiction.

In conclusion, we have presented a method called joint‐atlas‐optimization to improve the accuracy of multiatlas segmentation, especially at the distal part of the anatomical structure. We evaluated it in segmentation of the complex LA structure, including LA chamber, PVs, and LAA. Trained and tested on the public LA benchmark database, the proposed method showed improved performance over the best in the Benchmark in terms of both accuracy and robustness. In particular, it produced significantly improved segmentation accuracy in the PVs and LAA, the small distant part of LA geometry that is intrinsically difficult to follow by the conventional pairwise atlas approach. Accurate segmentation of LA chamber, PVs, and LAA potentially provides clinically interesting reference for guidance and evaluation of the interventional procedures in AF patients.
